# Lifestyle Factors Associated with Perceived Stress Among Adolescents: The Indirect Role of Mediterranean Diet Adherence in the Screen Time–Stress Association

**DOI:** 10.3390/healthcare14162621

**Published:** 2026-08-19

**Authors:** Pedro Delgado-Floody, Cristian Alvarez, Claudio Hernández-Mosqueira, Guido Contreras-Diaz, Indya Del-Cuerpo, Mauricio Cresp-Barria, Jordan Hernandez-Martínez, Pablo Valdés-Badilla, Cristian Núñez-Espinosa, Carlos Arriagada-Hernández, Gerardo Fuentes-Vilugrón, Felipe Caamaño-Navarrete

**Affiliations:** 1 Department of Physical Education, Sport and Recreation, Universidad de La Frontera, Temuco 4811230, Chile; pedro.delgado@ufrontera.cl; 2 Exercise and Rehabilitation Sciences Institute, Faculty of Rehabilitation Sciences, Universidad Andres Bello, Santiago 7591538, Chile; cristian.alvarez@unab.cl; 3 Departamento de Ciencias de la Educación, Universidad del Bio-Bio, Chillan 3780000, Chile; chernandez@ubiobio.cl; 4 Escuela de Kinesiología, Facultad de Ciencias de la Rehabilitación y Calidad de Vida, Universidad San Sebastián, Lago Panguipulli 1390, Puerto Montt 5501842, Chile; guido.contreras@uss.cl; 5 Department of Physical Education and Sports, Faculty of Sport Sciences, Sport and Health University Research Institute (iMUDS), University of Granada, 18012 Granada, Spain; delcuerpo@ugr.es; 6 Departamento de Educación e Innovación, Universidad Católica de Temuco, Temuco 4780000, Chile; mcresp@uct.cl; 7 Department of Physical Activity Sciences, Universidad de Los Lagos, Osorno 5290000, Chile; jordan.hernandez@ulagos.cl; 8 Department of Education, Faculty of Humanities, Universidad de la Serena, La Serena 1700000, Chile; 9 Department of Physical Activity Sciences, Faculty of Education Sciences, Universidad Católica del Maule, Talca 3460000, Chile; valdesbadilla@gmail.com; 10 Sports Coach Career, Faculty of Life Sciences, Universidad Viña del Mar, Viña del Mar 2520000, Chile; 11 Escuela de Medicina, Universidad de Magallanes, Punta Arenas 6200000, Chile; cristian.nunez@umag.cl; 12 Red Interuniversitaria de Envejecimiento Saludable de Latinoamérica y Caribe (RIES-LAC), Chile; 13 Physical Education Career, Universidad Autónoma de Chile, Temuco 4780000, Chile; carlos.arriagada@uautonoma.cl; 14 Facultad de Educación, Universidad Católica de Temuco, Temuco 4780000, Chile; gerardo.fuentes@uct.cl; 15 Facultad de Salud, Universidad Santo Tomás, Temuco 4780000, Chile

**Keywords:** mediterranean diet, screen time, adolescents, stress, mental health, mediation analysis

## Abstract

**Highlights:**

**What are the main findings?**

- Elevated screen time directly correlates with higher adolescent perceived stress.
- The mediterranean diet acts as a partial mediator in the link between screen time and perceived stress.

**What are the implications of the main findings?**

- School health programs must replace isolated screen-reduction targets with multifaceted behavioral interventions.
- Given the heightened psychological vulnerability found in adolescents with poor behavioral profiles, educational establishments must deploy targeted health strategies to support emotional regulation.

**Abstract:**

Background: Lifestyle habits are critical determinants of psychological health. This study aimed to determine the association between lifestyle factors and perceived stress levels, examining the potential mediating role of Mediterranean diet adherence (MDA). Methods: A cross-sectional study was conducted with 1229 participants (50.04% female, mean age of 13.45 ± 1.97 years). Lifestyle factors, including MDA, screen time (ST), sleep duration, active commuting and physical activity, were assessed. A multiple linear regression and mediation analysis were performed, adjusting for age and sex. Results: Significant sex differences were found, with females reporting higher stress and lower MDA (*p* < 0.001). Regression models revealed that higher MDA (*p* = 0.001) and lower ST (*b* = 0.22, *p* = 0.014) were significantly related to reduced stress. Mediation analysis showed that MDA statistically (partially) mediated the association between ST and stress (Indirect effect = 0.025, 95%CI; 0.005, 0.054). Specifically, MDA accounted for 10.12% of the total effect of ST on stress, representing a modest indirect contribution. Conclusions: Elevated ST routines are positively associated with adolescent perceived stress, whereas higher MDA displays an inverse relationship with stress. Additionally, this association is structurally modulated by MDA, highlighting that the unfavorable correlates of excessive television viewing and video-game use are modestly linked to concurrent variations in dietary quality within school environments. Given the cross-sectional design, these findings should be interpreted as indirect associations rather than causal pathways, and reverse or bidirectional relationships cannot be ruled out.

## 1. Introduction

Mental health is a cornerstone of the human experience. Beyond the mere absence of clinical illness, it is fundamentally defined as a multidimensional state of well-being that enables individuals to realize their potential, navigate daily stressors, work productively, and contribute meaningfully to their communities [1]. Mental health disorders, particularly perceived stress, have become a leading cause of global disease burden [2], correlating with a decreased quality of life during adolescence [3]. Notably, this behavioral and emotional strain is mirrored at the neurobiological level, as adolescent development involves critical structural maturation of the amygdala, hippocampal formation, and prefrontal cortex. Existing evidence highlights that these specific limbic and cortical regions are highly sensitive to chronic stress, suggesting that lifestyle-related distress may disrupt structural brain development and alter emotional regulation during this developmental window [4].

Recent evidence suggests that contemporary lifestyle factors are associated with psychological well-being [5]. In particular, modern behavioral patterns, often characterized by a high reliance on digital devices and technology, are increasingly linked to poor mental health outcomes and psychological vulnerability in school-aged and adolescent population [6,7].

Among these modern digital behaviors, elevated screen time (ST) has emerged as a major variable associated with psychological distress [6], where excessive daily ST routines are consistently tied to heightened emotional strain, sleep disruptions, and perceived stress in adolescent cohorts [8]. Evidence from a large-scale international survey encompassing over 191,000 adolescents demonstrated that prolonged ST, regardless of the typology, is concurrently linked to heightened school stress. Specifically, daily screen exposure exceeding four hours particularly computer use and electronic gaming was consistently associated with significantly higher odds of psychological strain [6]. Longitudinal evidence has also connected extreme screen exposure to severe psychological distress, showing that high daily ST is concurrently associated with an increased risk of severe clinical outcomes such as suicidal ideation and behavior in both sexes. These observations reinforce the idea that elevated screen time is consistently associated with long-term adverse indicators of emotional well-being during adolescence [5].

The literature indicates that the relationship between technological overexposure and psychological strain may not operate in isolation; rather, it is deeply intertwined with variations in daily dietary behaviors. In this context, Mediterranean diet adherence (MDA) characterized by a high intake of plant-based foods, healthy fats, and antioxidants displays a robust inverse relationship with mental distress [9]. Previous research has shown that high dietary quality is concurrently associated with lower psychiatric symptoms and better overall mental health dimensions in schoolchildren [10]. Consistently, a cross-sectional investigation utilizing the Krece Plus quick test (which corresponds to the KIDMED index, the Mediterranean Diet Quality Index for children and adolescents) among 698 European adolescents demonstrated that higher MDA is significantly and inversely related to psychological distress, displaying a robust relationship with lower odds of experiencing depressive symptoms [11]. This evidence reinforces the relevance of assessing MDA within modern lifestyle frameworks, further validating its position as a critical correlate of adolescent emotional well-being and a key variable in concurrent behavioral models.

Supporting this framework, a systematic review synthesizing data from over 3000 children and adolescents confirmed a significant inverse relationship between MDA and psychiatric indicators, particularly depression and anxiety [12]. This robust evidence further supports the inclusion of MDA as a key variable within pediatric and adolescence epidemiology, justifying its evaluation as a structurally linked factor to adolescent mental health outcomes. Furthermore, a systematic review pooling data from 6796 pediatric subjects confirmed a significant, positive cross-sectional correlation between higher MDA and optimal health-related quality of life metrics [10]. Therefore, an optimal dietary quality represents a pivotal correlate not only of isolated psychological symptoms but also of the broader multidimensional framework of adolescent subjective well-being.

Similarly, large-scale evidence indicates that regular physical activity practice is significantly associated with optimal sleep quality metrics within a complex framework concurrently tied to perceived stress [13]. Hence, physical activity and sleep duration as critical lifestyle variables that must be accounted for when examining the overarching statistical relationships between screen-related behaviors, dietary patterns, and adolescent mental health. Furthermore, current pediatric data demonstrate that active commuting (e.g., walking or cycling to school) displays a significant inverse association with stress metrics, whereas female sex is consistently tied to higher susceptibility to psychological distress. These insights highlight that active commuting represents an additional relevant lifestyle variable within the behavioral framework that concurrently links screen overexposure, dietary patterns, and adolescent perceived stress [14].

While it is established that sedentary screen-based routines often coexist with unfavorable eating habits, the precise underlying pathways connecting these behaviors within specific emotional domains remain poorly understood. Specifically, it remains unclear whether the relationship between ST and adolescent perceived stress is indirectly associated with or partially explained by variations in MDA, a mechanism that can be quantified through structural statistical mediation. Therefore, this study aimed to determine the association between lifestyle factors and perceived stress levels, examining the potential mediating role of MDA. Consistently, we hypothesized that the direct association between elevated ST and heightened perceived stress is partially mediated by MDA, indicating an indirect behavioral pathway linking screen exposure to psychological distress.

## 2. Materials and Methods

### 2.1. Study Design and Participants

This investigation adhered to the Strengthening the Reporting of Observational Studies in Epidemiology (STROBE) statement guidelines for cross-sectional research [15]. The target population consisted of secondary school students recruited through convenience (non-probabilistic) sampling from subsidized educational centers located in urban sectors of the Araucanía Region, Chile. The definitive cohort comprised 1229 adolescents, exhibiting an equal distribution between sexes: 614 males (49.96%) and 615 females (50.04%).

An a priori statistical power calculation was executed via G*Power software (v. 3.1.9.7) to ensure adequate parameter stability for the multivariable linear regression and mediation analysis [16]. To rigorously detect even minor or subtle behavioral and psychological interactions without capitalizing on chance, the statistical parameters for an F-test were set under highly stringent criteria: a small anticipated effect size (f^2^ = 0.025), a strict significance alpha level of 0.05 (alpha = 0.05), and an exceptionally high statistical power of 95% (1 − beta = 0.95), accounting for the 7 total predictors and covariates included in the fully adjusted model. Based on these mathematical configurations, a minimum sample size of 884 participants was established as the mandatory threshold to avoid type II errors and ensure stable parameter estimations. Therefore, the final enrollment of 1229 adolescents substantially exceeded this demanding baseline, providing an optimal and highly sensitive statistical framework to evaluate the structural pathways linking ST exposure, MDA, and perceived stress.

Eligible participants were students with active enrollment in the selected schools. Exclusion criteria applied were (i) any documented physical or medical condition preventing the understanding or execution of the protocols, and (ii) absence on data collection days or incomplete informed consent documentation.

The study design strictly followed the ethical standards of the Declaration of Helsinki (2013) and received formal authorization from the Institutional Review Board/Ethics Committee of the Universidad Autónoma de Chile (Registry: CEC 13-25). Voluntary student assent and signed written informed parental/guardian consent were mandatory requirements before data collection.

**Figure 1:**
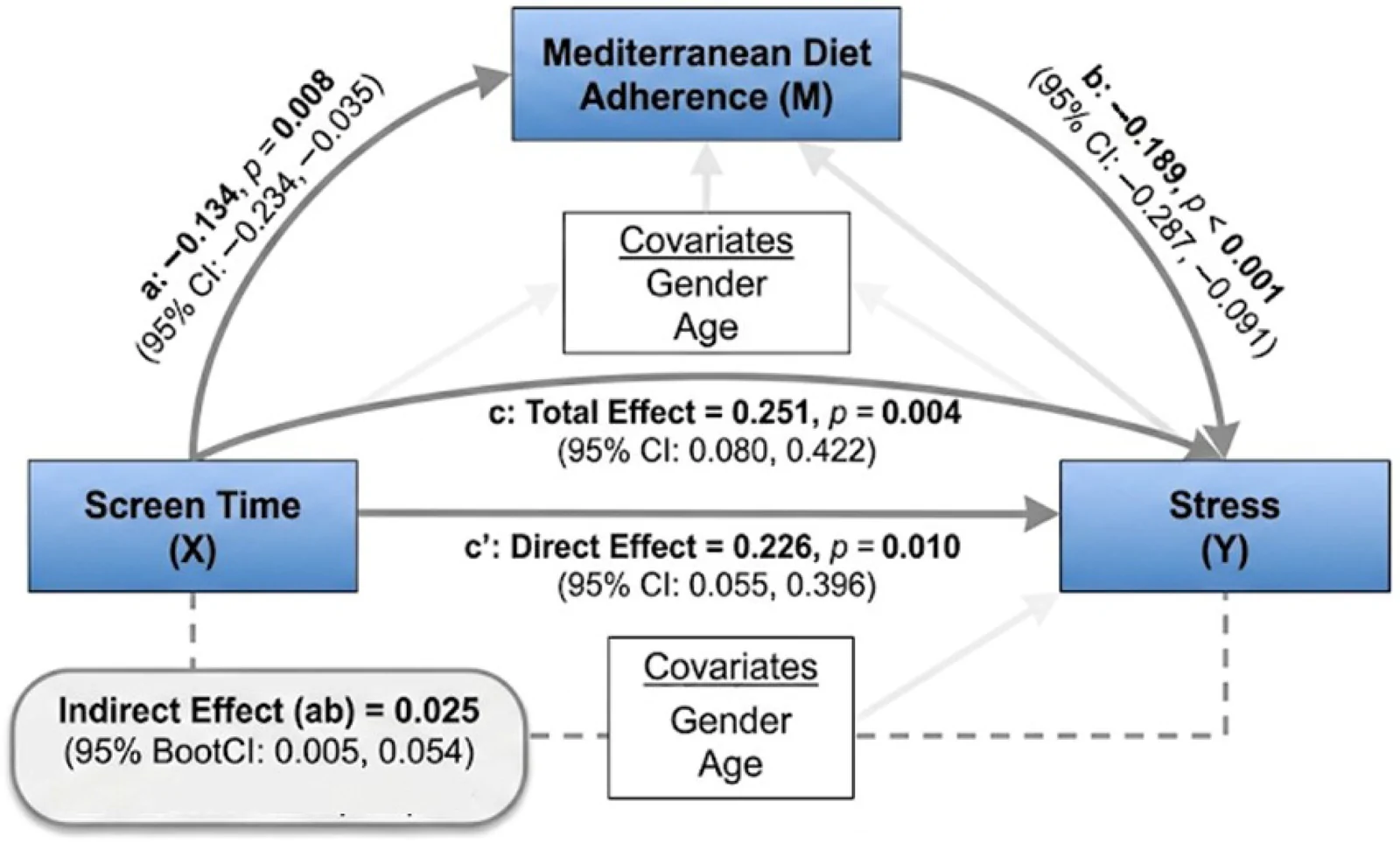
Mediation model testing whether the association between screen time and stress was mediated by Mediterranean diet adherence. Note: The proportion mediated (10.12%) was calculated using exact unrounded model parameters (ab/c) × 100 and serves as a supplementary descriptive index.

Disclosure of Generative AI Use: During the preparation of this manuscript, the authors used Gemini 1.5 Pro (Google) to assist in refining the conceptual translation of technical results into English, restructuring and paraphrasing arguments within the Discussion section to minimize redundancy with prior publications, and designing the structural layout and infographic presentation of the mediation pathways illustrated in Figure 1. Following the use of this tool, the authors critically reviewed, verified, and edited all outputs, and take full responsibility for the data integrity, scientific accuracy, and final content of the manuscript.

### 2.2. Procedures

Data collection took place within the schools during regular class hours using digital, self-administered questionnaires. Before starting, researchers briefed the students on the study’s scope, emphasizing that participation was strictly voluntary, entirely confidential, and that they could withdraw at any time without consequences.

#### 2.2.1. Perceived Stress

Psychological strain was quantified using the stress subscale of the short-form Depression, Anxiety, and Stress Scale (DASS-21) [17]. Although this 21-item, multidimensional self-report instrument is originally designed to measure three distinct emotional domains depression, anxiety, and stress [18,19,20], only the stress subscale was processed and analyzed in the present study to strictly align with the established research objectives. The stress subscale consists of 7 items evaluating levels of irritability, persistent tension, difficulty relaxing, and overthinking during the previous week. Each item is scored on a 4-point Likert scale ranging from 0 (‘did not apply to me at all’) to 3 (‘applied to me very much or most of the time’), yielding a subscale score range from 0 to 21 points, where lower values represent lower levels of perceived stress. For characterization purposes, psychological stress was classified according to standard clinical cut-offs: mild (8–9 points), moderate (10–12 points), severe (13–16 points), and extremely severe (17 or more points) [21]. The DASS-21 has been previously validated in Chilean student populations [22] demonstrating excellent psychometric behavior, brevity, and ease of administration [23], as well as optimal internal consistency in adolescent cohorts (Cronbach’s alpha > 0.80) [24]. It is worth noting that while instruments like Cohen’s Perceived Stress Scale (PSS) capture global cognitive appraisals of stress over a 30-day period, the DASS-21 stress subscale specifically captures operationalized symptoms of chronic affective arousal, tension, and perceived emotional distress during the past week.

#### 2.2.2. Mediterranean Diet Adherence (MDA)

The Krece Plus quick test (which corresponds to the KIDMED index, the Mediterranean Diet Quality Index for children and adolescents) was implemented to assess adherence to the Mediterranean dietary pattern through eating habits and overall dietary quality. Throughout this manuscript, MDA therefore refers to the degree of adherence to a Mediterranean-type dietary pattern captured by this index rather than to a generic nutritional-risk score [25]. Designed to detect nutritional and physical risks linked to youth obesity, this index includes dichotomous (yes/no) questions scored as +1 or −1. The total score is classified based on previous recommendations as follows: (i) 8 to 12: Good food habits; (ii) 4 to 7: Moderate food habits (indicating a need for dietary improvement); and (iii) 0 to 3: Poor food habits (indicating very low diet quality) [25]. This tool has been previously validated in student populations [26].

#### 2.2.3. Lifestyle

Screen time and physical activity

Sedentary screen-based behaviors and active habits were captured using the PA Krece Plus test. This brief survey records the average daily hours dedicated to television or video games (i.e., ST) and the weekly frequency of after-school PA. Accordingly, in the present study “screen time” refers specifically to recreational television viewing and video-game use and does not capture other contemporary forms of screen exposure such as smartphone use, social media, tablets, streaming, or school-related digital activities; this operational definition should be considered when interpreting the findings [27]. The classification is made according to the number of hours for each item. To ensure the index is logically interpretable where a higher total score reflects a healthier lifestyle ST hour were reverse-coded as follows: 5 points: <1 h/day, 4 points: 1 h/day, 3 points: 2 h/day, 2 points: 3 h/day, 1 point: 4 or more hours/day. Conversely, points for PA were assigned directly (higher frequency resulting in higher points).

The total points are added up, and the person is accordingly classified as having either a good lifestyle (male ≥ 9 h, female ≥ 8 h), a regular lifestyle (male 6–8 h, female 5–7 h), or a bad lifestyle (male ≤ 5 h, female ≤ 4 h). The questionnaires were completed individually by the adolescents in the presence of evaluators.

#### 2.2.4. Active Commuting

School travel patterns were evaluated using the self-reported PACO (Pedalea y Anda al COle) questionnaire, developed by the University of Granada and validated for Chilean youth [28,29]. This tool specifically measures walking and cycling habits to and from educational centers in secondary school students and has been previously utilized in Chilean cohorts [30]. Following standard protocols, participants reported their daily weekly transit via two targeted questions: “How do you go to school?” and “How do you come back from school?”. Modes of transport were dichotomized into “active” (walking or cycling) or “non-active” (car or bus) commuting. The psychometric robust character of this questionnaire has been confirmed, demonstrating high reliability within Chilean schoolchildren sub-samples.

#### 2.2.5. Sleep Duration

Habitual sleep patterns were captured via self-report. Participants stated the average number of hours they slept per night during regular school days. This continuous variable is widely accepted in pediatric epidemiological research to estimate sleep duration.

### 2.3. Statistical Analysis

All the statistical analysis were performed with SPSS statistical software version 23.0 (SPSS^TM^ Inc., Chicago, IL, USA) and the PROCESS macro (version 4.1, creator Andrew F. Hayes, Calgary, Alberta, Canada) developed by Hayes. Statistical significance was set at *p* < 0.05 for all tests. Initially, data cleaning was conducted to identify missing values and outliers. The distribution of continuous variables was evaluated using the Kolmogorov–Smirnov test alongside visual inspection of histograms and Q-Q plots. Although slight deviations from normality were observed in certain continuous lifestyle metrics, parametric tests were applied given the large sample size (n > 1200), supported by the Central Limit Theorem regarding the normality of sampling distributions. Beyond this, key diagnostic assumptions for multivariable linear regressions were systematically verified: linearity and homoscedasticity were confirmed via inspection of standardized residual plots against predicted values, approximate normality of regression residuals was checked, and influential observations were screened using Cook’s distance (D < 1.0). The initial sample comprised 1229 adolescents who provided baseline descriptive data. For the multivariable linear regressions and mediation models, a complete-case analysis approach was adopted. Missing values across individual questionnaire items accounted for less than 12% of total data (147 participants had missing data on at least one specific covariate or lifestyle predictor), yielding a final analytical sample of n = 1082 complete cases with no statistical imputation required.

Descriptive statistics were calculated, expressing continuous variables as Mean ± Standard Deviation (SD) and categorical variables (active commuting) as absolute frequencies and percentages. To examine sex differences, independent samples t-tests were used for continuous outcomes, and a Chi-square test (chi^2^) was applied for the categorical variable (active commuting). To evaluate the simultaneous association of lifestyle factors with *p* stress, multivariable linear regression models were constructed. Model 1 examined the unadjusted associations of lifestyle variables (MDA, ST, sleep duration, PA, and active commuting) with stress. Model 2 was adjusted for demographic variables (age and sex) to account for potential confounding and baseline differences. Multicollinearity among predictors was checked using the Variance Inflation Factor (VIF < 1.074), confirming the absence of multicollinearity in the regression models. To evaluate the explanatory power of the proposed models, we calculated the coefficient of determination (R^2^).

To explore the mechanism linking ST to stress, a mediation analysis was conducted using the PROCESS macro (Model 4). To preserve parsimony and focus on the direct behavioral pathway, the mediation model specifically controlled for age and sex as demographic covariates. The indirect effect (ab) was evaluated using a non-parametric bootstrapping procedure with 5000 resamples. An indirect effect was considered statistically significant if its 95% bias-corrected bootstrap confidence interval (95% CI) did not encompass zero. Finally, the proportion of the total effect explained by the mediator was calculated using the formula: (ab/c) × 100, where ab represents the indirect effect and c represents the total effect [31].

## 3. Results

### 3.1. Sample Characteristics

The descriptive characteristics of the sample and the comparison by sex are summarized in Table 1. The mean age of the participants was 13.45 ± 1.97 years, with no significant differences observed between male and female (*p* = 0.793), indicating a well-balanced sample regarding age distribution. Significant sex differences were found in the psychological and lifestyle variables. Stress scores were significantly higher in females compared to males (10.24 ± 5.20 vs. 8.59 ± 4.73, respectively; *p* < 0.001). Regarding lifestyle habits, males exhibited significantly higher MDA (*p* < 0.001) and a higher frequency of daily PA (*p* < 0.001). Furthermore, although ST was significantly higher in males (3.37 ± 1.70 vs. 3.05 ± 1.64 h/d; *p* = 0.001), they also reported a longer sleep duration (8.03 ± 1.44 vs. 7.80 ± 1.58 h/d; *p* = 0.010) than their female counterparts. These findings suggest that sex is a determining factor in both the perceived stress levels and the lifestyle choices of the study population.

### 3.2. Multiple Linear Regression Analysis

The hierarchical regression analysis revealed significant associations with stress among the participants (see Table 2). In Model 1, MDA was negatively associated with stress levels (*b* = −0.19, *p* < 0.001), indicating that higher dietary quality is linked to lower stress. Similarly, active commuting showed a significant negative association with the dependent variable stress (*b* = −0.89, *p* = 0.004). After adjusting for demographic variables (sex and age), MDA and active commuting remained with a significant association (*p* = 0.001 and *p* = 0.003 respectively). In addition, ST reported a positive association with stress *(b* = 0.22, *p* = 0.014), suggesting that increased sedentary ST is associated with higher stress scores. In contrast, sleep duration and PA h/day did not reach statistical significance in either model (*p* > 0.05). Finally, the initial model explained 3.0% of the variance (R^2^ = 0.030) which increased to 6.1% when adjusted for age and sex (R^2^ = 0.061).

### 3.3. Mediation Analysis

A mediation analysis was performed using the PROCESS macro (Model 4) to examine whether MDA mediated the relationship between ST and stress, while controlling for sex and age (see Figure 1). The results indicated that ST had a significant negative association with MDA (a = −0.134, *p* = 0.008). In turn, MDA had a significant negative association with stress levels (b = −0.189, *p* < 0.001). The total effect of ST on stress was significant (c = 0.251, *p* = 0.004). When MDA was included in the model, the direct effect of ST on stress remained significant (c′ = 0.226, *p* = 0.010), suggesting partial mediation. Furthermore, the indirect effect of ST on stress through MDA was statistically significant (Effect = 0.025; SE = 0.013; 95% CI [0.005, 0.054]). Since the bootstrap confidence interval does not include zero, it can be concluded that MDA partially explains the statistical association between increased ST and higher stress levels. The mediation analysis showed that MDA accounted for 10.12% of the total effect of ST on stress levels (Table 3). This indicates that while ST has a significant direct association with stress, a portion of this association is explained by the unfavorable dietary patterns associated with high screen exposure.

## 4. Discussion

This study aimed to determine the association between lifestyle factors and perceived stress, examining the potential mediating role of MDA. The findings of the present study expand the contemporary understanding of lifestyle medicine by illuminating the intricate pathways connecting ST, MDA, and adolescent psychological distress. Concurrently, multivariable linear regression analysis confirmed that lifestyle choices are significantly linked to psychological well-being (i.e., perceived stress), demonstrating that while MDA presented a robust negative association with distress, elevated ST was significantly and positively associated with adolescent perceived stress. Interestingly, the association between ST and stress reached statistical significance only after adjusting for demographic covariates (model 2). This pattern points to a statistical suppression effect driven primarily by sex. As male adolescents reported higher daily ST but lower baseline stress than female peers, unadjusted estimates conflated these inverse sex trends. Controlling for sex accounted for this variance, unmasking the direct positive association between screen exposure and psychological distress.

Additionally, the mediation analysis demonstrating that increased ST exposure is unfavorably associate with emotional outcomes, a relationship that is formally mediated by a concurrent variation in MDA. Although the indirect path through MDA was statistically significant, its practical magnitude was modest (accounting for ~10.12% of the total effect, or ~0.025 points on the DASS-21 stress subscale). This suggests that while dietary quality represents a relevant co-occurring behavioral factor, screen time influences adolescent perceived stress predominantly through direct pathways or other unmeasured lifestyle and psychosocial mechanisms.

In this context, The results of the present study are consistent with a recent large-scale network analysis of 52,782 students, which demonstrated that modern screen-related habits, such as elevated ST, are directly intertwined with core symptoms of agitation and overreaction within adolescent psychological distress network [32]. While our study identified a robust direct relationship between high ST routines and perceived stress during adolescence, evidence from younger US pediatric cohorts reveals a nuanced, age-dependent divergence where screen exposure remains unassociated with well-being indicators in toddlers but severely elevates behavioral vulnerabilities in preschool children [33]. This contrast suggests that although the psychological correlates of excessive screen exposure spans across development, its specific manifestation shifts from externalizing behavioral disruptions in early childhood to standardized domains of perceived stress and emotional strain during adolescence. Supporting these observations, a systematic review confirmed that excessive ST, particularly through smartphone and weekday social media use, is consistently associated with diminished psychological well-being in adolescents [34].

According to findings reported in the literature, adolescents who exhibit high patterns of screen use systematically experience higher perceived stress scores and affective regulation difficulties compared to those with more balanced habits. Beyond adolescent behavioral risks, evidence associates excessive and addictive screen media exposure with severe physiological alterations, including cortisol dysregulation, heightened sympathetic arousal, and poor stress regulation [35]. Therefore, data from previous studies support that cumulative ST burden acts as a priority environmental risk predictor, positively correlating with an increase in susceptibility to distress and psychological exhaustion within the current educational environment [36].

In this context, identifying dietary behaviors that may be associated with reduced screen-related vulnerabilities becomes paramount. Specifically, the results of the present study reinforce previous evidence regarding the critical role of lifestyle factors in adolescent mental health, while substantially advancing the literature through the identification of a significant explanatory mechanism: MDA acts as a partial mediator in the relationship between excessive ST and perceived stress. The results suggest that the protective effect of MDA against stress does not rely exclusively on concurrent healthy behaviors, but rather stems from intrinsic neurobiological mechanisms linked to its nutritional profile [37]. Specifically, key components of MDA such as omega-3 fatty acids, B vitamins, and polyphenols exert robust antioxidant and anti-inflammatory actions that enhance emotional regulation and strengthen neurological resilience to modern environmental stressors, including excessive screen exposure [38].

In this regard, evidence has highlighted that MDA is positively related to mental health variables in the school context [39] with a higher compliance to this dietary pattern being associated with a lower risk of developing psychological symptoms, even after controlling for sociodemographic and lifestyle variables [40]. While growing evidence has linked the onset of different mental health problems to lifestyle factors such as diet quality, further research has been demanded to rigorously establish causal inferences beyond traditional cross-sectional associations [41].

Our most notable finding advances this field by identifying a significant indirect pathway, demonstrating that the psychological toll of modern digital habits such as ST is statistically associated with nutritional behaviors, although this indirect pathway was modest and does not establish causality. Instead of examining lifestyle components in isolation [38,42], the mediation framework reveals that a degradation in MDA partially explains how excessive ST translates into heightened perceived stress. From a physiological and school-setting standpoint, considering dietary quality is highly relevant for the educational system [10] as a better MDA is systematically associated with lower levels of distress [41]. This protective mechanism may be explained by the synergistic action between key nutritional components of the MDA and the neurobiological systems that regulate autonomic arousal and somatic manifestations of stress [43]. From an applied standpoint, adherence to the Mediterranean pattern is itself shaped by modifiable behavioral determinants: nutrition knowledge and related literacy have been shown to translate into more sustainable eating and higher Mediterranean diet adherence [44]. Conversely, screen-based environments may also relate to eating behavior, as patterns of social media use have been associated with emotional and maladaptive eating in some populations [45]; these links, however, remain heterogeneous and were not directly tested in the present study.

Finally, the descriptive and comparative findings of this study revealed significant sex-based differences within this behavioral framework, showing that while female adolescents exhibited significantly higher levels of perceived stress, their male peers reported higher compliance with healthy lifestyle behaviors, including greater MDA, PA levels, and sleep duration. Crucially, this localized trend is strongly reinforced by broader systematic evidence highlighting a heightened female vulnerability to screen-related risks, which directly aligns with our observed sex differences and direct screen-to-stress pathways [34]. This cross-cultural convergence not only underscores the robustness of our results but also emphasizes the critical need to examine how modern screen devices interact with concurrent lifestyle variables within the adolescent environment to modulate psychological health. Notably, the recent literature indicates that healthy lifestyle patterns, particularly higher MDA and regular PA, are inversely linked to psychological strain, displaying a favorable relationship with emotional regulation and stress reactivity metrics in female populations [7]. However, the consistent asymmetry in distress levels among females underscores the relevance of sex-sensitive approaches that integrate nutritional, behavioral, and psychosocial dimensions to promote emotional regulation during adolescence.

Concurrently, in the present study active commuting (e.g., walking or cycling to school) displays a significant inverse association with adolescent perceived stress metrics. This finding aligns with emerging pediatric data highlighting that active transportation is associated with lower psychological distress in school-aged populations [14]. Our findings regarding the psychological benefits of active commuting align with a large-scale evidence from 21,596 Chinese children and adolescents. In that cohort, active commuting to school significantly predicted lower odds of experiencing depressive symptoms compared to passive transport [46]. This cross-cultural convergence reinforces that the emotional benefits of active travel transcend geographic environments, supporting active transportation as a behavior associated with lower distress. Furthermore, within the school ecology, walking or cycling to educational establishments provides adolescents with a transitional period of environmental interaction and physical detachment from sedentary screen-based routines, which may support emotional regulation and psychological resilience before entering the classroom environment. While systematic evidence indicates that active commuting to school may not directly alter cognitive performance or academic achievement in youth, its primary value appears anchored in psychological well-being [47]. This distinction suggests that the neurological benefits of active travel are preferentially routed toward stress mitigation and emotional regulation rather than academic metrics.

### Limitations and Strengths

This study is limited by its cross-sectional design, which precludes temporal priority or definitive causal inferences regarding the observed associations, and its reliance on self-reported questionnaires susceptible to recall bias, which could underestimate or overestimate the responses. In addition, ST measurements were self-reported and may not fully reflect other significant sources of screen exposure among today’s adolescents—such as smartphones, social media, and computer use—so the results should be interpreted with caution. Several further limitations should be acknowledged. Because participants were recruited within schools, observations may be clustered at the school level; this clustering was not modeled in the present analyses and may have influenced the estimates, and should be regarded as a limitation. In addition, the regression and mediation models were adjusted only for age and sex, so relevant confounders—including socioeconomic status, parental education, body mass index, pubertal stage, sleep quality, mental health history, the household food environment, and academic pressure—were not accounted for, and residual confounding is therefore likely. Consequently, and given the cross-sectional design, the reported associations should be interpreted with caution and not as evidence of causal mediation. Conversely, a major strength of this study is its robust sample size, which provided adequate statistical power.

## 5. Conclusions

This study demonstrates that digital lifestyle choices and nutritional behaviors are significantly and concurrently associated with psychological well-being during adolescence. The findings verify that elevated ST routines are positively associated with perceived stress, whereas a higher MDA is inversely tied to emotional strain. Crucially, mediation analysis confirms that this relationship between ST and perceived stress is partially and statistically mediated by MDA, indicating that the unfavorable correlates of excessive television viewing and video-game use are modestly linked to variations in dietary quality; given the cross-sectional design, this should be read as an indirect association rather than a causal mechanism.

The present investigation contributes to a better understanding of how adolescents’ daily habits relate to the stress they experience. Our findings suggest that more screen time is associated with higher levels of perceived stress. Although the cross-sectional design provides only a snapshot and does not allow us to establish cause and effect, the results underscore the importance of promoting healthy routines from an early age and placing emphasis on adolescents’ psychological well-being.

## Figures and Tables

**Table 1:** Descriptive statistics and comparative analysis of stress and lifestyle factors by sex.

Variables	Male (n = 614)	Female (n = 615)	Total (n = 1229)	*p*-Value _(F)_ *t*-Test
	Mean ± SD	Mean ± SD	Mean ± SD	
Age (years)	13.43 ± 1.98	13.46 ± 1.96	13.45 ± 1.97	0.793 _(0.07)_
Stress (score)	8.59 ± 4.73	10.24 ± 5.20	9.42 ± 5.04	<0.001 _(33.84)_
MDA (score)	6.33 ± 2.92	5.50 ± 3.04	5.92 ± 3.01	<0.001 _(23.90)_
Screen Time (h/d)	3.37 ± 1.70	3.05 ± 1.64	3.21 ± 1.68	0.001 _(10.99)_
Sleep Duration (h/d)	8.03 ± 1.44	7.80 ± 1.58	7.92 ± 1.52	0.010 _(6.70)_
Physical Activity (h/d)	2.87 ± 1.97	2.32 ± 1.79	2.59 ± 1.90	<0.001 _(23.56)_
Active Commuting				chi^2^-test
Yes n (%)	253 (41.2%)	273 (44.4%)	526 (42.8%)	0.142
No n (%)	361 (58.8%)	342 (55.6%)	703 (57.2%)	

MDA = Mediterranean diet adherence. SD = Standard Deviation. F values are shown in parentheses. Differences between sexes were evaluated using independent samples *t*-tests for continuous variables and Chi-square (chi^2^) test for the categorical variable (active commuting).

**Table 2:** Association between lifestyle factors, sleep duration, active commuting and perceived stress.

	Model 1					Model 2		
	*b*	95% CI	β	*p*	*b*	95% CI	β	*p*
Mediterranean diet adherence	−0.19	[−0.30, −0.09]	−0.11	<0.001	−0.19	[−0.29, −0.08]	−0.11	0.001
Screen Time (hours)	0.17	[−0.01, 0.35]	0.06	0.065	0.22	[0.05, 0.40]	0.07	0.014
Sleep Duration (hours/day)	−0.13	[−0.33, 0.07]	−0.04	0.193	−0.15	[−0.34, 0.05]	−0.04	0.147
Physical Activity (hours/day)	−0.02	[−0.19, 0.14]	−0.01	0.79	0.04	[−0.12, 0.20]	0.14	0.645
Active Commuting	−0.89	[−1.50, −0.29]	−0.09	0.004	−0.92	[−1.51, −0.32]	−0.90	0.003
R^2^		0.030 (3.0%)				0.061 (6.1%)		
Model F Statistic ($df_{1},df_{2}$)	*F*(5, 1076) = 6.66, *p* < 0.001				*F*(7, 1074) = 9.91, *p* < 0.001			

Data shown represent *b*: Unstandardized regression coefficient and 95% confidence interval [95% CI]. β = standardized beta coefficient; CI = confidence interval; df = degrees of freedom. Model 1: Unadjusted model. Model 2: Adjusted for age and sex.

**Table 3:** Total, direct, and indirect effects summary.

Path	*b*	SE	t	*p*-Value	95% CI
Screen Time (ST) → MDA (a)	−0.134	0.051	−2.65	0.008	[−0.234, −0.035]
MDA → Stress (b)	−0.189	0.05	−3.788	<0.001	[−0.287, −0.091]
Screen Time → Stress (c′)	0.226	0.087	2.59	0.010	[0.055, 0.396]
Total Effect Model					
Screen Time → Stress (c)	0.251	0.087	2.874	0.004	[0.080, 0.422]
Indirect Effect					
ST → MDA → Stress	0.025	0.013	-		[0.005, 0.054]
%MEDIATION: 10.12%					

MDA = Mediterranean diet adherence. Note; *b* = unstandardized beta, CI = confidence interval; SE = standard error. Note: The proportion mediated (10.12%) was calculated using exact unrounded model parameters $\left(ab/c\right)\times 100$ and serves as a supplementary descriptive index.

## Data Availability

The data presented in this study are available on request from the corresponding author. The data are not publicly available due to privacy and ethical restrictions involving school-aged participants, as per the protocols approved by the Ethics Committee of Universidad Autónoma de Chile (ACTA; No. CEC 13-25).

## Abbreviations

The following abbreviations are used in this manuscript:

MDA	Mediterranean diet adherence
PA	Physical activity
ST	Screen time
